# Research on Extraction of Compound Fault Characteristics for Rolling Bearings in Wind Turbines

**DOI:** 10.3390/e22060682

**Published:** 2020-06-18

**Authors:** Ling Xiang, Hao Su, Ying Li

**Affiliations:** School of Mechanical Engineering, North China Electric Power University, Baoding 071003, China; suhao19970418@163.com (H.S.); LiYing021298@163.com (Y.L.)

**Keywords:** rolling bearing, fault detection, multi-point optimal minimum entropy deconvolution adjusted (MOMEDA), 1.5-dimensional Teager kurtosis spectrum, wind turbine

## Abstract

Wind turbines work in strong background noise, and multiple faults often occur where features are mixed together and are easily misjudged. To extract composite fault of rolling bearings from wind turbines, a new hybrid approach was proposed based on multi-point optimal minimum entropy deconvolution adjusted (MOMEDA) and the 1.5-dimensional Teager kurtosis spectrum. The composite fault signal was deconvoluted using the MOMEDA method. The deconvoluted signal was analyzed by applying the 1.5-dimensional Teager kurtosis spectrum. Finally, the frequency characteristics were extracted for the bearing fault. A bearing composite fault signal with strong background noise was utilized to prove the validity of the method. Two actual cases on bearing fault detection were analyzed with wind turbines. The results show that the method is suitable for the diagnosis of wind turbine compound faults and can be applied to research on the health behavior of wind turbines.

## 1. Introduction

As a renewable and clean energy source worldwide, wind energy has gradually received increasing attention. However, the working environment of the wind turbine is poor, and the variable load fluctuation makes the wind turbine component more easily damaged [1]. Therefore, to guarantee wind turbine safe operation it is important to carry out timely failure identification by applying running condition data [2,3]. As the main components and parts of rotating machinery, rolling bearings are widely applied in wind turbines [4,5]. In the actual operation of the wind turbine faults often do not occur separately, and one fault often causes other faults to occur. When multiple faults occur and their fault features are coupled, this kind of fault is called a composite fault [6,7]. Compared with a single fault, in composite faults the characteristics of different components are mixed with each other, and the interference between them adds to the difficulty of fault extraction [8]. Therefore, how to effectively diagnose the bearing compound fault is still a hot issue [9,10]. Generally, there are two main difficulties in the diagnosis of composite fault: (1) the pulse components generated by different faults are often overwhelmed in the time domain waveform; and (2) different faults may produce the same or different resonant frequencies [11].

Vibration signal analysis is the preferred method for diagnosing bearing faults [12]. When the rolling bearing of wind turbine fails, the fault signal is often accompanied by the occurrence of periodic shock components [13,14]. In this case, it can be used to identify the frequency of impact components in the vibration signal to detect the bearing fault. Since the measured signal is obtained by convolution of the periodic shock signal and resonance response of mechanical components, deconvolution is able to recover periodic pulses [15]. Currently, the application of minimum entropy deconvolution (MED) is extensively used. The MED method mainly enhances the pulse component in the original signal by maximizing the kurtosis of the filtered signal [16]. MED is generally applied to extract fault features from raw vibration signals with large amounts of noise. Endo and Randall [17] used MED and autoregressive models to form a new deconvolution technique and verified its effectiveness through gearbox vibration signals. Jiang et al. [18] proposed a diagnosis method for weak rolling bearing faults founded on MED and the envelope spectrum. Mcdonald et al. [19] studied and concluded that the MED algorithm could preferentially deconvolute a single pulse instead of repeating the required periodic pulses during the fault, so maximum correlation kurtosis deconvolution (MCKD) was proposed. The effectiveness of the simulation signal was verified by comparing it with the vibration signal of the gearbox. However, MCKD is an iterative process, and the selected filter is not optimal and is limited by too many model parameters and complex resampling processes [20]. In response to the limitations of MED and MCKD in rotating machinery applications, Mcdonald and Zhao [21] developed an improved deconvolution method, which was the multipoint optimal minimum entropy deconvolution adjusted (MOMEDA). MOMEDA has improved the definition of deconvolution for the characteristics of rotating machinery fault signals, and introduces the target vector and multi-point D-norm to provide a non-iterative optimal solution. In this algorithm, continuous pulses are obtained by multi-point kurtosis deconvolution, which is made available for periodic fault feature extraction. Therefore, when the components in the bearing system fail, the impact components associated with each component failure have their own cycles, and the MOMEDA algorithm can separate the desired signal source by setting the corresponding deconvolution cycle. Therefore, MOMEDA was made available for bearing compound fault diagnosis. As an envelope demodulation method, the 1.5-dimensional Teager kurtosis spectrum with good suppression is used to effectively demodulate the amplitude-modulated signal. Thus, a new approach was proposed here based on MOMEDA and the 1.5-dimensional Teager kurtosis spectrum to extract composite fault features for wind turbines. A bearing composite fault simulation signal with strong background noise was utilized to prove the validity of the method. Two actual cases from wind turbines were analyzed to detect the faults of rolling bearings. The proposed method can effectively detect the composite faults of wind turbines.

The rest of this paper is organized as follows: In Section 2 and Section 3, the MOMEDA and the 1.5-dimensional Teager kurtosis spectrum algorithms are reported. In Section 4, we describe the implementation process of bearing composite fault separation and extraction of MOMEDA and the 1.5 dimension Teager kurtosis spectrum. The validity and usefulness of the method is presented by simulations and examples in Section 5. This article is summarized in Section 6.

## 2. Multipoint Optimal Minimum Entropy Deconvolution Adjusted (MOMEDA)

Deconvolution is founded on the definition of a signal metric, commonly known as a norm. A problem with the MED solution is that it is an iterative selection process, and will not necessarily design an optimal filter for the posed problem [21]. MOMEDA is a new non-iterative deconvolution method which is used to deconvolute the composite fault signal. MOMEDA is applied in non-integer fault periods, and there is no resampling stage.

Suppose $\vec{y}$ is a shock signal of a faulty bearing, $\vec{h}$ represents the frequency response function, $\vec{x}$ stands for collected vibration signal, and $\vec{e}$ represents random noise. Then, the transmission process of the impulse signal from the signal source to the sensor can be approximated as Equation (1) [19]:

(1) $$\vec{x}=\vec{y}*\vec{h}+\vec{e}$$

The core part of MOMEDA algorithm aims to obtain an optimal filter $\vec{f}$ in a non-iterative way to reconstruct the original fault impact signal $\vec{y}$ and minimize the influence of noise on extracted impact signal. The deconvolution process is Equation (2):

(2) $$\vec{y}=\vec{f}*\vec{x}=\sum_{k=1}^{N-L}f_{k}x_{k+L-1}$$

where the value of k is 1,2,⋯,N−L. The MOMEDA algorithm extracts the multi-point D-norm based on the D-norm for the characteristics of periodic shock in the rotating machinery fault signal. The multipoint D-norm is written as Equations (3) and (4) by [21]:

(3) $$\mathrm{Multi}\;D\text{-}\mathrm{Norm}=MDN(\vec{y},\vec{t})=\frac{1}{\left\|\vec{t}\right\|}\frac{{\vec{t}}^{T}\vec{y}}{\left\|\vec{y}\right\|}$$

(4) $$MOMEDA:\underset{\vec{f}}{\max}\;MDN(\vec{y},\vec{t})=\underset{\vec{f}}{\max}\frac{{\vec{t}}^{T}\vec{y}}{\left\|\vec{y}\right\|}$$

where $\vec{t}$ represents the target vector, which indicates the location and weight of the impact component of the convolution target.

When using MOMEDA for multi-fault detection, the failure period in vibration signal should be considered Equation (5):

(5) $$\begin{array}{l} t_{n}=P_{n}(T)=\delta_{\mathrm{round}(T)}+\delta_{\mathrm{round}(2T)}+\cdots ,\\ \vec{t}=\vec{P}(T) \end{array}$$

where $t_{n}$ stands for the pulse at signal n, and T represents the deconvolution period.

When the target vector $\vec{t}$ is completely matched with the original impact signal $\vec{y}$, the deconvolution effect is optimal. At this time, the multi-point D-norm obtains the maximum value, and the corresponding filter is a set of optimal filter $\vec{f}$.

Solving the problem of Equation (4) is equivalent to solving the Equation (6):

(6) $$\frac{d}{d\vec{f}}\left(\frac{{\vec{t}}^{T}\vec{y}}{\left\|\vec{y}\right\|}\right)=0$$

where

(7) $$\vec{f}=f_{1},f_{2},\cdots ,f_{L},\vec{t}=t_{1},t_{2},\cdots ,t_{N-L}$$

The Equation (8) can be obtained from Equations (2), (4), and (6):

(8) $$\frac{d}{d\vec{f}}\left(\frac{{\vec{t}}^{T}\vec{y}}{\left\|\vec{y}\right\|}\right)={\left\|\vec{y}\right\|}^{-1}(t_{1}{\vec{M}}_{1}+t_{2}{\vec{M}}_{2}+\cdots +t_{K}{\vec{M}}_{K})-{\left\|\vec{y}\right\|}^{-3}{\vec{t}}^{T}\vec{y}X_{0}\vec{y}=0$$

If $X_{0}=\left[M_{1},M_{2},\cdots ,M_{k}\right]$, then Equation (8) will be abbreviated as Equation (9):

(9) $${\left\|\vec{y}\right\|}^{-1}X_{0}\vec{t}-{\left\|\vec{y}\right\|}^{-3}{\vec{t}}^{T}\vec{y}X_{0}\vec{y}=\vec{0}$$

It can be written as Equation (10):

(10) $$\frac{\vec{t}\cdot \vec{y}}{{\left\|\vec{y}\right\|}^{2}}X_{0}\vec{y}=X_{0}\vec{t}$$

When $\vec{y}=X_{0}{}^{T}\vec{f}$ is brought into Equation (10), this can be represented as Equation (11):

(11) $$\frac{{\vec{t}}^{T}\vec{y}}{{\left\|\vec{y}\right\|}^{2}}\vec{f}={\left(X_{0}X_{0}{}^{T}\right)}^{-1}X_{0}\vec{t}$$

Give a particular solution to Equation (11), and it can be recorded as Equation (12):

(12) $$\vec{f}={\left(X_{0}X_{0}{}^{T}\right)}^{-1}X_{0}\vec{t}$$

Where Equation (13):

(13) $$X_{0}={\left[\begin{array}{ccccc} x_{L} & x_{L+1} & x_{L+2} & \cdots & x_{N}\\ x_{L-1} & x_{L} & x_{L+1} & \cdots & x_{N-}{}_{1}\\ x_{L-2} & x_{L-1} & x_{L} & \cdots & x_{N-2}\\ \cdots & \cdots & \cdots & \cdots & \cdots \\ x_{1} & x_{2} & x_{3} & \cdots & x_{N-L-r+1} \end{array}\right]}_{L\times N-L+1}$$

Substituting Equation (12) into $\vec{y}=X_{0}{}^{T}\vec{f}$, the original shock signal $\vec{y}$ can be restored.

## 3. The 1.5-Dimensional Teager Kurtosis Spectrum

The k-order cumulant of the zero-mean stationary random signal x(n) is defined as Equation (14) by [22]:

(14) $$c_{kx}(\tau_{1},\tau_{2},\cdots ,\tau_{k-1})=E\left[x(n)x(n+\tau_{1})\cdots x(n+\tau_{k-1})\right]-E\left[g(n)g(n+\tau_{1})\cdots g(n+\tau_{k-1})\right]$$

where g(n) is a Gaussian random composition with the same second-order statistic as x(n). From this this definition, the higher-order cumulant can not only measure the high-order correlation of the time series, but also reflect the degree of the stochastic process away from the Gaussian distribution. That is, the non-Gaussian of the signal can be measured. The high-order cumulant of Gaussian noise is zero, so the high-order cumulant can suppress the noise influence well and improve the analysis and recognition accuracy.

The third-order cumulant expression of x(n) can be derived from the definition of higher-order cumulants as Equation (15):

(15) $$c_{3x}=E\left[x(n)x(n+\tau_{1})x(n+\tau_{2})\right]$$

Here, $\tau_{1}=\tau_{2}=\tau$ can be taken to get the diagonal slice of the third-order cumulant as Equation (16):

(16) $$c_{3x}=E\left[x(n)x(n+\tau )x(n+\tau )\right]$$

The 1.5-dimensional spectrum is defined as a one-dimensional Fourier transform of this diagonal slice Equation (17):

(17) $$B(\omega )=\int_{-\infty }^{+\infty }c_{3x}(\tau ,\tau )\;e^{-j\omega \tau }d\tau$$

The 1.5-dimensional spectrum is obtained by the Fourier transform of high-order cumulants, which can restrain noise well and analyze nonlinear and non-Gaussian signals effectively [22].

Teager kurtosis [23] as a fourth-order statistic can reflect signal departure from Gaussian distribution and characterize impact signal characteristics. The sliding Teager kurtosis method is utilized to abstract periodic impact components of the signal in this paper. The sliding Teager kurtosis method mainly obtains different sliding Teager kurtosis time series by changing the calculation time length of Teager kurtosis. The sliding Teager kurtosis is defined as Equation (18) by [23]:

(18) $$C(t_{i})=c_{4y}\left[x(i),x(i+L-1)\right],\;i=1,2,\cdots ,n$$

where $C(t_{i})$ is the ith sample point of the sliding Teager kurtosis time series, and $c_{4y}[\cdot ]$ represents the value of the Teager kurtosis of [x(i),x(i+L−1)]. $c_{4y}[\cdot ]$ is taken into the absolute value. Due to noise and other factors, L is generally in the range 2≤L≤15.

By calculating the 1.5-dimensional spectrum for M(n) (the sliding Teager kurtosis time series) with the largest kurtosis value, the 1.5-dimensional Teager kurtosis spectrum for x(n) can be obtained as Equation (19):

(19) $$B(\omega )=\int_{-\infty }^{+\infty }c_{3M}(\tau ,\tau )\;e^{-j\omega \tau }d\tau$$

The 1.5-dimensional Teager kurtosis spectrum is provided, with high-order cumulants and excellent properties in the Teager energy operator. The method can suppress noise well, track the instantaneous energy change, and reflect the non-Gaussian characteristics. When the rolling bearing of the wind turbine fails, the vibration signal departure from Gaussian distribution arises due to the phenomenon of amplitude modulation. Therefore, the method can realize bearing fault diagnosis in wind turbines, demodulating fault characteristic frequency successfully and extracting the weak shock fault characteristics of the bearing.

## 4. Fault Feature Extraction Process

When a composite fault occurs in a rolling bearing of a wind turbine, it is often accompanied by a large amount of noise interference. The source signals from multiple faults are mixed with each other, causing great obstacles in the detection of faults. The key to compound fault diagnosis is whether it can effectively separate the fault characteristic frequencies corresponding to different damage components. The fault components related to different component faults in the signal have their own periods. In the MOMEDA algorithm, the deconvolution period of interest can be input. Other sources will be defaulted to noise components, and the deconvolution process can separate the desired signal sources. Therefore, the MOMEDA algorithm is suitable for processing composite fault signals of wind turbine bearings with periodic shock and low signal-to-noise ratio characteristics.

When the bearing system fails, the picked-up vibration signal has amplitude and frequency modulation, and a departure of impact signal arises from the Gaussian distribution. The wind turbine fault signal is analyzed by the 1.5-dimensional Teager kurtosis spectrum, which can suppress noise well and demodulate the characteristic frequency of the bearing fault.

**Figure 1:**
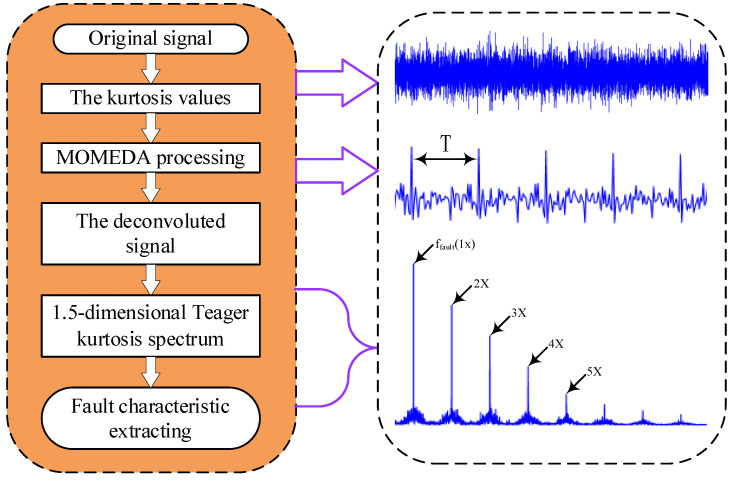
Flow chart of extraction method founded on multi-point optimal minimum entropy deconvolution adjusted (MOMEDA) and 1.5-dimensional Teager spectrum.

In summary, to achieve accurate discrimination of composite faults, the advantages of MOMEDA and the 1.5-dimensional Teager kurtosis spectrum are combined for bearing fault detection for wind turbines. The proposed extraction method of composite fault feature is presented in Figure 1.

The specific implementation process is as follows:

(1) First, MOMEDA preprocessing is performed by setting deconvolution periods of different faults;

(2) Then, 1.5-dimensional Teager kurtosis spectrum analysis is performed on the deconvolved signal preprocessed by MOMEDA;

(3) According to bearing fault frequency and the results in previous step, the type of composite fault for the bearing is detected.

## 5. Case Analysis

### 5.1. Case 1

To prove the validity of the novel extraction method, in this section a case is presented where bearing composite faults were separated and diagnosed by the new method for wind turbines from the National Renewable Energy Laboratory (NREL). For this case, a 750-KW wind turbine gearbox with a high-speed shaft bearing was provided; the model was the SKF32222 J2 tapered roller bearing. The gearbox body was installed with the roller bearing in the radial position of the high-speed shaft. The sampling frequency was set to 40 kHz. The rotating speed of the shaft was 1800 r/min. After the end of the experiment, the gearbox was disassembled and the high-speed shaft bearing had suffered severe wear damage and overheating. The high-speed shaft frequency of the gearbox $f_{r}$, the high-speed gear meshing frequency $f_{m1}$, the medium-speed gear meshing frequency $f_{m2}$, the inner ring defect frequency $f_{i}$ of the high-speed rolling bearing, and the holder defect frequency $f_{c}$ are all displayed in Table 1.

The kurtosis values (See Table 2) of the sliding Teager kurtosis time series were calculated according to the deconvolved inner ring and holder signals. These kurtosis values were obtained for different sliding length L conditions. They are represented using K1 and K2, respectively. K1 represents the kurtosis value of the bearing inner ring source signal. K2 is the kurtosis value of the bearing hold source signal.

**Figure 2:**
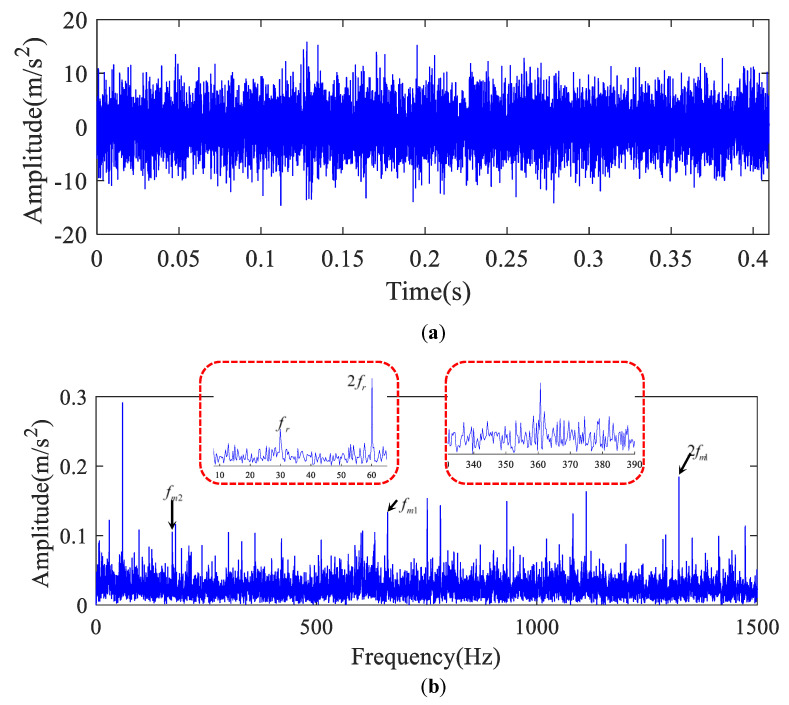
Original signal of a high-speed shaft bearing for a wind turbine. (**a**) The time series and (**b**) its envelope spectrum.

**Figure 3:**
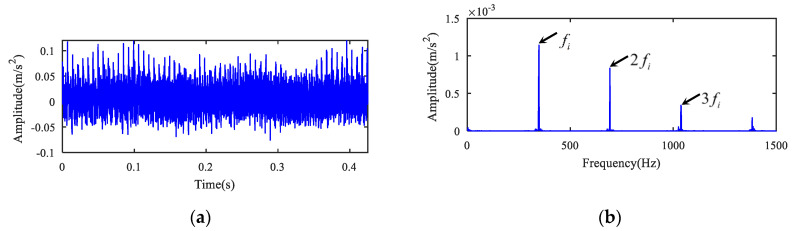
The analysis of the inner ring fault signal for case 1. (**a**) The deconvoluted signal time domain and (**b**) its 1.5-dimensional Teager kurtosis spectrum.

The original signal is indicated in Figure 2a when the rotating speed was about 30 Hz in the high-speed shaft. The envelope spectrum and 1.5-dimensional Teager kurtosis spectrum of composite signal are presented in Figure 2b and Figure 3b. In Figure 2b, the high-speed shaft frequency components $f_{r}$ and $2f_{r}$, high-speed gear meshing frequency $f_{m1}$, and the medium-speed gear meshing frequency $f_{m2}$ are found. However, there were no prominent defect frequency components.

The measured original signal (see Figure 2a) was analyzed. The MOMEDA algorithm was first utilized to deconvolute the original signal, and the deconvolution period was set to the inner ring fault period $T_{i}=f_{s}/f_{i}=115.8$. Then, 1.5-dimensional Teager kurtosis spectrum analysis was performed. The deconvoluted time signal and its 1.5-dimensional Teager kurtosis are indicated in Figure 3a,b. The sliding length L was set to 2 because the kurtosis value (K1) (See Table 2) of the sliding Teager kurtosis time series for inner ring fault signal was the largest. Figure 3b displays the result of 1.5-dimensional Teager kurtosis spectrum analysis when the sliding window length was 2. From Figure 3b, it can be seen that the fault frequency $f_{i}$ and $2f_{i},\;3f_{i},\;\cdots$ of the bearing inner ring were accurately extracted. Noise was suppressed. There was no other defect frequency component, and the fault frequency of bearing $f_{i}$ was effectively separated and presented at the same time using the novel extraction method.

**Figure 4:**
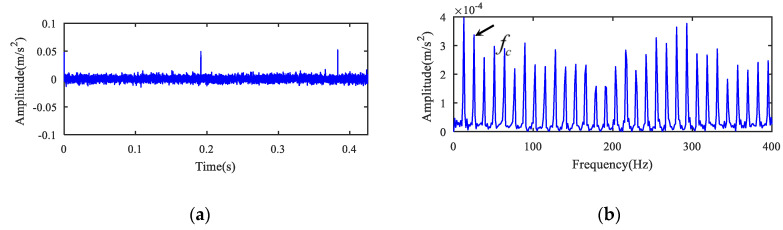
The holder fault signal analysis for case 1. (**a**) The deconvoluted signal and (**b**) its 1.5-dimensional Teager kurtosis spectrum.

The analysis results of holder fault signal are displayed in Figure 4. MOMEDA algorithm was first used to deconvolute the original signal, and the deconvolution period was set to the holder failure period $T_{c}=f_{s}/f_{c}=3137.3$. Then, 1.5-dimensional Teager kurtosis spectrum analysis was performed. The deconvoluted time signal and its 1.5-dimensional Teager kurtosis are demonstrated in Figure 4a,b. The sliding length L was set to 2 because the kurtosis value (K2) (see Table 2) of the sliding Teager kurtosis time series for the holder fault signal was the largest. Figure 4b displays the result of 1.5-dimensional Teager kurtosis spectrum analysis when the sliding window length was 2. In Figure 4b, the fault frequency $f_{c}$ of the bearing holder and harmonics were accurately extracted. Noise was suppressed, and there was no other defect frequency component. According to the above analysis results, it can be concluded that the bearing inner ring and holder were faulty at the same time, and the two faults were separated and diagnosed effectively by the novel method.

**Figure 5:**
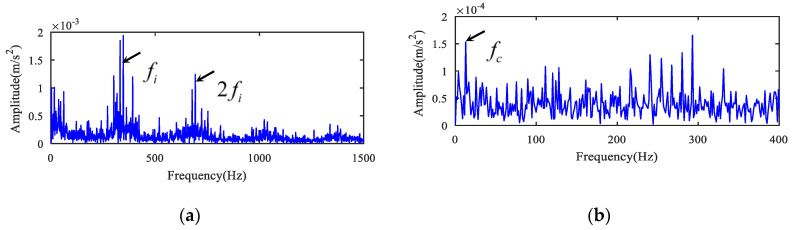
Envelope spectrum analysis results for the deconvolved signals of case 1. (**a**) Envelope spectrum of the deconvolved signal for the inner ring fault. (**b**) Envelope spectrum of the deconvolved signal for the holder fault.

Envelope spectrum analysis was performed directly on the deconvolved signals in Figure 3a and Figure 4a. The analysis results are demonstrated in Figure 5a,b. The fault frequency of the bearing inner ring $f_{i}$ is extracted in Figure 5a, and the fault frequency $f_{c}$ of bearing holder is also demonstrated in Figure 5b. However, the characteristics are not clear and prominent due to background noise as compared with Figure 3b and Figure 4b. The two faults were separated and detected effectively by combining MOMEDA with the 1.5-dimensional Teager kurtosis spectrum, as demonstrated in Figure 3b and Figure 4b. The synthetical method can effectively eliminate redundant interference lines and suppress background noise.

**Figure 6:**
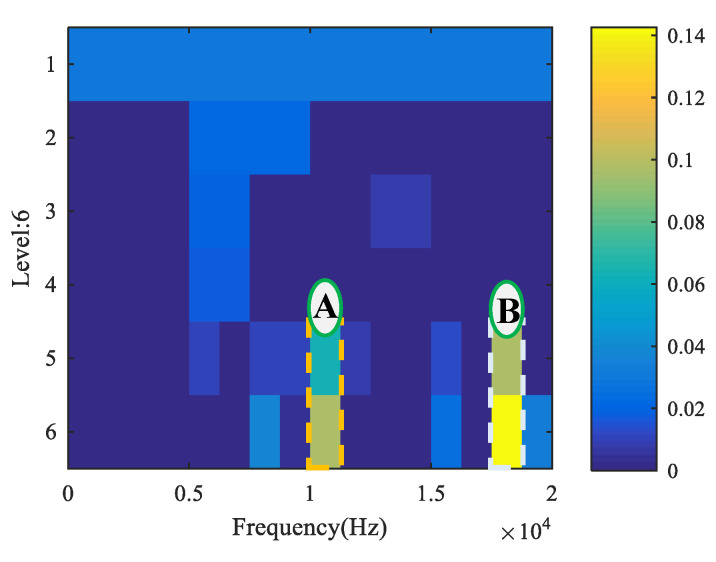
Kurtogram of measured signal for case 1.

**Figure 7:**
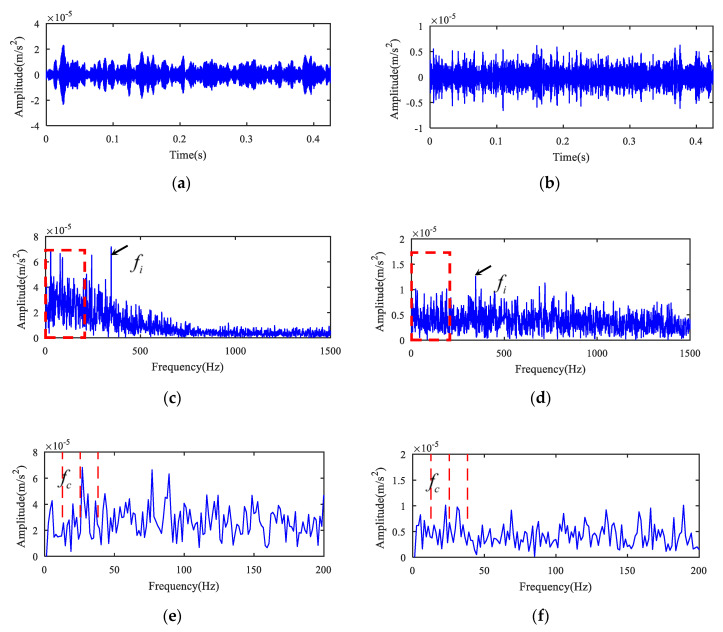
Results using SK + minimum entropy deconvolution (MED) for case 1. (**a**) Filtered signal after band A by MED. (**b**) Filtered signal after band B by MED. (**c**) Envelope spectrum of (**a**). (**d**) Envelope spectrum of (**b**). (**e**) Partially enlarged view of (**c**). (**f**) Partially enlarged view of (**d**).

In addition, for the sake of illustrating the superiority of the novel method, the measured signals were analyzed and compared by the spectral kurtosis (SK) and MED method referred to in [15]. The corresponding kurtogram is shown in Figure 6. There are two resonant frequency bands. The resonant frequency band A was from 10,000 Hz to 11,250 Hz and the resonant frequency band B was from 17,500 Hz to 18,750 Hz. The original signal was filtered with band A and band B to obtain the filtered signal, and the filtered signal was handled by the MED method. Figure 7a,c,e present the analysis of frequency band A, and Figure 7b,d,f present the analysis of frequency band B. As can be concluded from Figure 7c,d, the fundamental frequency components of the inner race fault were prominent, but the background noise interference was very large and the cage fault feature could not be displayed, even in enlarged spectrums as demonstrated in Figure 7e,f. The SK and MED method could not separate the composite fault features, but the novel method was able to eliminate interference successfully and extract fault features, as demonstrated in Figure 3b and Figure 4b. The comparison between MED and the proposed method is presented in Table 3.

### 5.2. Case 2

**Figure 8:**
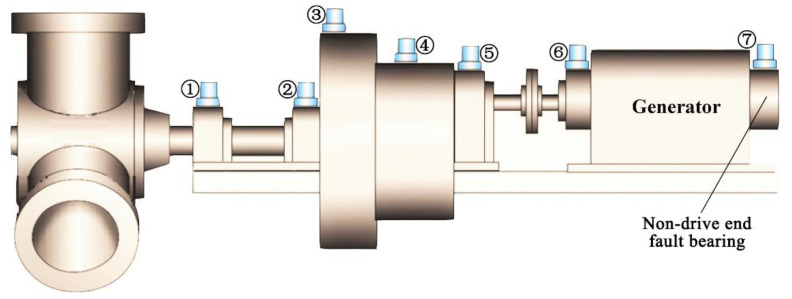
Wind turbine drive system.

Another case is presented where bearing composite faults were separated and diagnosed by the novel method in wind turbine from a wind farm set in Hebei, China. The wind turbine used a three-stage gearbox. The first stage was a planetary gear, and the second and the third stages (middle stage and high speed stage) were parallel helical gears. The acceleration measurement was adopted during vibration testing. Figure 8 illustrates the structural diagram of wind turbine drive system. The condition monitoring system (CMS) was applied to obtain data on the gearbox faults. The structure sketch and the exact placement of the sensor of a gearbox for wind turbine are shown in Figure 8. There were seven sensors installed in the gearbox system. The fault occurred in the seventh sensor, as shown in Figure 8. That is, the experimental data were measured by the vibration acceleration sensor on the generator. Output shaft frequency was $f_{r}=21.6$ Hz. The bearing model was SKF 6330M.C3 (deep groove ball bearing). The feature frequencies of the SKF 6330M.C3 bearing are illustrated in Table 4.

**Figure 9:**
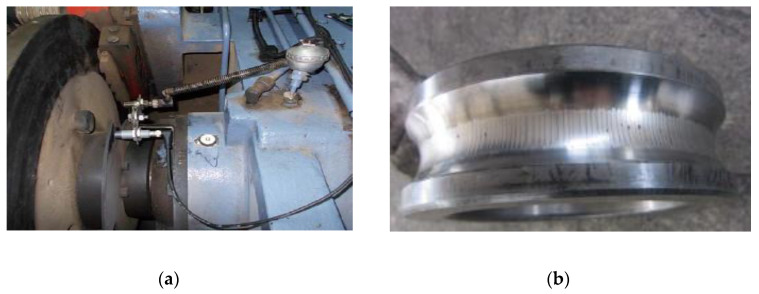
The testing pictures. (**a**) The transducer in the gearbox and (**b**) a bearing fault on the inner race.

**Figure 10:**
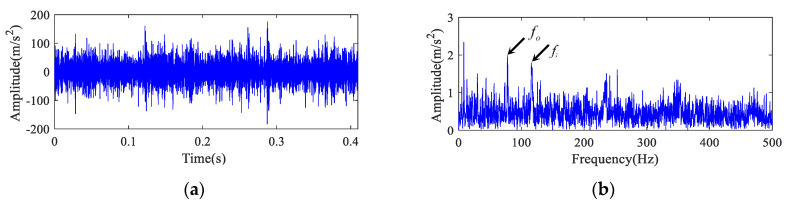
Raw signal of non-drive end bearing of the generator. (**a**) The time series and (**b**) its envelope spectrum.

The test picture is displayed in Figure 9. A transducer in the gearbox is shown in Figure 9a. The bearing fault on inner ring is presented in Figure 9b. The time domain waveform and envelope spectrum of the fault bearing vibration signal are presented in Figure 10. As shown in Figure 10b, the fundamental frequency of bearing inner and outer rings defect frequency could be extracted, but their harmonic frequencies could not be presented. Therefore, the diagnosis of bearing fault type is difficult to achieve.

Table 5 displays kurtosis values of the sliding Teager kurtosis time series for the deconvolved outer ring and inner signals. These kurtosis values are obtained for different sliding length L conditions. They are represented using K1 and K2, respectively. K1 is the kurtosis value of outer ring source signal. K2 is the kurtosis value of inner ring source signal.

**Figure 11:**
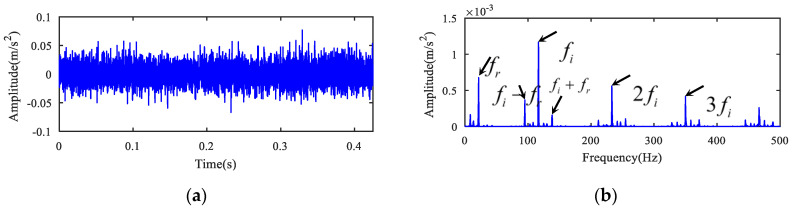
The analysis of bearing inner ring fault signal for case 2. (**a**) The time series and (**b**) its 1.5-dimensional Teager kurtosis spectrum.

The measured original fault signal (see Figure 10a) was analyzed. The MOMEDA algorithm was first utilized to deconvolute the original signal, and the deconvolution period was set to the inner ring fault period $T_{i}=f_{s}/f_{i}=140.39$. Then, 1.5-dimensional Teager kurtosis spectrum analysis was performed. The deconvoluted time signal and its 1.5-dimensional Teager kurtosis are displayed in Figure 11a,b. The sliding length L was set to 2 because the kurtosis value (K1) (See Table 5) of the sliding Teager kurtosis time series for the inner ring fault signal was the largest when L = 2. Figure 11b displays the result of 1.5-dimensional Teager kurtosis spectrum analysis when the sliding window length was 2. From Figure 11b, it can be seen that the inner race failure frequency, its doubling frequency component, and the modulation frequency components of the characteristic frequency were clearly extracted. From the spectrum, it can be concluded that the noise was evidently suppressed. There was no other defect frequency component, and the feature frequency of bearing inner ring fault was effectively separated and presented at the same time utilizing the novel method.

**Figure 12:**
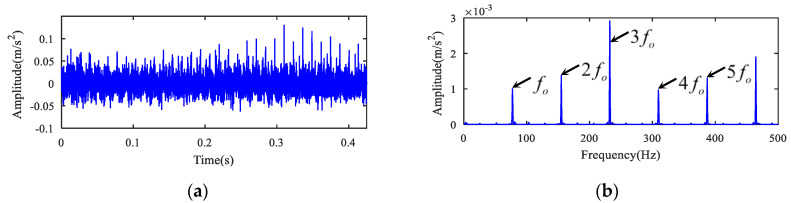
The analysis of the outer ring fault signal for case 2. (**a**) The time series and (**b**) its 1.5-dimensional Teager kurtosis spectrum.

The analysis results from the bearing outer ring fault are displayed in Figure 12. The MOMEDA algorithm was first utilized to deconvolute the original signal, and the deconvolution period was set to the outer ring failure period $T_{o}=f_{s}/f_{o}=211.68$. Then, 1.5-dimensional Teager kurtosis spectrum analysis was performed. The deconvoluted time signal and its 1.5-dimensional Teager kurtosis are displayed in Figure 12a,b. Sliding length L was set to 3 because the kurtosis value (K2) (see Table 4) of the sliding Teager kurtosis time series for the outer ring fault signal was the largest when L = 3. The result of 1.5-dimensional Teager kurtosis spectrum analysis is presented in Figure 12b when the sliding window length was 3. In Figure 12b it can be seen that the fault frequency $f_{o}$ of outer ring and its harmonic frequencies were accurately extracted. The spectrum shows that the noise was suppressed, and there was no other defect frequency component. According to the above analysis results, it appears that bearing inner and outer rings were faulty at the same time, and the two faults were separated and detected effectively by utilizing the novel method. These diagnosis results are consistent with the actual situation.

**Figure 13:**
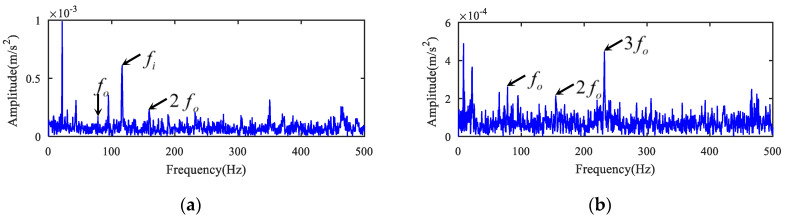
Envelope spectrum analysis results for the deconvolved signals of case 2. (**a**) Envelope spectrum of the deconvolved signal for inner ring fault; (**b**) Envelope spectrum of the deconvolved signal for outer ring fault.

Envelope spectrum analysis was performed directly on the deconvolved signals in Figure 11a and Figure 12a, and the results are illustrated in Figure 13a,b. The failure frequency of bearing inner ring $f_{i}$ is presented in Figure 13a. Besides, the outer ring failure frequency $f_{o}$ and its double frequency component are with lower amplitudes in Figure 13a. This means that the separation of the inner race failure feature was insufficient. The envelope spectrum of the outer race fault is displayed in Figure 13b. It was found that the amplitudes of the extracted outer race fault $f_{o}$ and 2$f_{o}$ were all low. The fault features in Figure 13b were not clear due to background noise as compared with Figure 11b. The two faults were separated and detected effectively when combining MOMEDA with the 1.5-dimensional Teager kurtosis spectrum, as illustrated in Figure 11b and Figure 12b. The synthetical method can effectively eliminate redundant interference and extract the fault characteristics.

**Figure 14:**
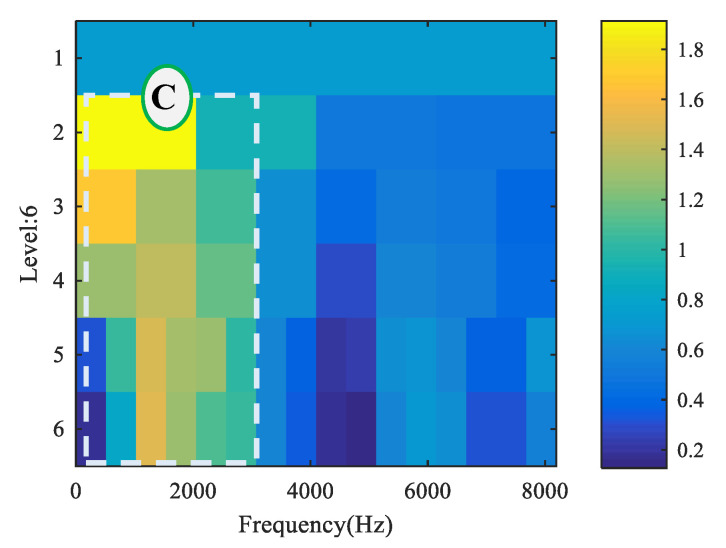
Kurtogram of the measurement signal for case 2.

**Figure 15:**
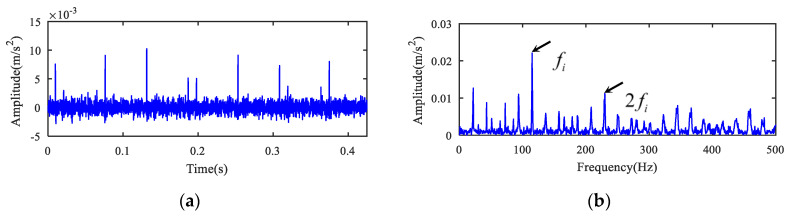
Results using SK + MED for case 1. (**a**) Filtered signal after band C by MED. (**b**) Envelope spectrum of (**a**).

The kurtogram of measured original fault signal is presented in Figure 14. Only one resonant frequency band, marked as band C, was observed in the kurtogram. The MED analysis result is displayed in Figure 15. As shown in Figure 15b, only the inner race fault characteristic frequency $f_{i}$ and its double component could be extracted, but no relevant component of outer race fault was found. The spectrum was interspersed with a number of interference lines. It is hard to decide the type of bearing compound fault accurately, but the novel method is capable of eliminating interference and extracting fault features effectively, as displayed in Figure 11b and Figure 12b. The comparison between MED and the proposed method for case 2 is presented in Table 6.

## 6. Conclusions

The paper proposes a novel method based on MOMEDA and the 1.5-dimensional Teager kurtosis spectrum for bearing compound fault diagnosis in wind turbines. Using this method, MOMEDA was utilized to deconvolute the compound fault signal as a preprocessing application. The kurtosis values of sliding Teager kurtosis time series were computed for different bearing fault signals. The sliding length was selected for 1.5-dimensional Teager kurtosis spectrum analysis, which was then was performed and was aimed at obvious fault information. Synthetic analyses of simulated and actual monitoring signals show that the new method can restrain strong background noise well and achieve accurate separation of compound fault features for rolling bearings. When comparing the proposed method with other methods, the proposed method is capable of eliminating interference and separating the composite fault features, and the features of different faults are extracted effectively. The results prove that this new method has apparent strengths in the compound fault diagnosis of rolling bearings for wind turbines

## Figures and Tables

**Table 1:** Gearbox fault characteristic frequency.

Name	$f_{r}$	$f_{m1}$	$f_{m2}$	$f_{i}$	$f_{c}$
Frequency/Hz	30	660	172.5	345.3	12.75

**Table 2:** The kurtosis values of the sliding Teager kurtosis time series for case 1.

L	2	3	4	5	6	7	8	9	10	11	12	13	14	15
K1	132.10	122.80	118.70	100.42	79.45	67.26	58.75	52.66	49.92	46.49	42.87	41.32	40.20	38.59
K2	2904.2	2106.1	1655.2	1316.7	942.0	1206.0	1360.2	866.4	611.4	494.6	429.3	385.6	353.1	327.5

**Table 3:** The comparison of the methods for case 1.

	Fault Features	Advantages	Disadvantages
SK + MED	Invisible and noisy	Prominent fundamental frequency	Cannot separate the composite fault features
MOMEDA	Visible and clear	Eliminating interference	Can separate composite fault features

**Table 4:** Test bearing parameters.

Bearing Type	SKF 6330M.C3
Inner ring failure frequency	116.7 Hz
Outer ring failure frequency	77.4 Hz
Rolling element failure characteristic frequency	51 Hz
Cage failure characteristic frequency	8.6 Hz

**Table 5:** The kurtosis values of the sliding Teager kurtosis time series for case 2.

L	2	3	4	5	6	7	8	9	10	11	12	13	14	15
K1	84.00	82.88	74.38	57.36	43.19	37.32	37.14	38.08	38.41	38.42	38.42	38.72	39.19	39.05
K2	417.12	452.85	311.59	216.84	151.71	101.80	67.33	45.69	33.13	27.96	27.00	28.38	30.44	31.94

**Table 6:** The comparison of the methods for case 2.

	Fault Features	Advantages	Disadvantages
SK + MED	Visible inner race fault frequency	Prominent fundamental frequency	Cannot separate the composite fault features
MOMEDA	Visible inner and outer race fault frequencies	Eliminates interference	Can separate composite fault features
